# The 100 Most Cited Papers About Cancer Epigenetics

**DOI:** 10.7759/cureus.7623

**Published:** 2020-04-10

**Authors:** Ignacio Jusue-Torres, Joshua E Mendoza, Malcolm V Brock, Alicia Hulbert

**Affiliations:** 1 Neurosurgery, Loyola University Chicago, Stritch School of Medicine, Chicago, USA; 2 Sugery, University of Illinois at Chicago, Chicago, USA; 3 Surgery, Johns Hopkins University School of Medicine, Baltimore, USA; 4 Surgery, University of Illinois at Chicago, Chicago, USA

**Keywords:** epigenetic, cancer, molecular biomarker, citation analysis, bibliometrics, dna methylation

## Abstract

Introduction

Although bibliometric analyses have been performed in the past on cancer and genomics, little is known about the most frequently cited articles specifically related to cancer epigenetics. Therefore, the purpose of this study is to use citation count to identify those papers in the scientific literature that have made key contributions in the field of cancer epigenetics and identify key driving forces behind future investigations.

Materials and methods

The Thomas Reuters Web of Science services was queried for the years 1980-2018 without language restrictions. Articles were sorted in descending order of the number of times they were cited in the Web of Science database by other studies, and all titles and abstracts were screened to identify the research areas of the top 100 articles. The number of citations per year was calculated.

Results

We identified the 100 most-cited articles on cancer epigenetics, which collectively had been cited 147,083 times at the time of this writing. The top-cited article was cited 7,124 times, with an average of 375 citations per year since publication. In the period 1980-2018, the most prolific years were the years 2006 and 2010, producing nine articles, respectively. Twenty-eight unique journals contributed to the 100 articles, with the Nature journal contributing most of the articles (n=22). The most common country of article origin was the United States of America (n=78), followed by Germany (n=4), Switzerland (n=4), Japan (n=3), Spain (n=2), and United Kingdom (n=2).

Conclusions

In this study, the 100 most-cited articles in cancer epigenetics were examined, and the contributions from various authors, specialties, and countries were identified. Cancer epigenetics is a rapidly growing scientific field impacting translational research in cancer screening, diagnosis, classification, prognosis, and targeted treatments. Recognition of important historical contributions to this field may guide future investigations.

## Introduction

In 1942, Conrad Hal Waddington was the first to use the Greek word “epigenesis”, to describe how cells differentiated, and thus epigenetics was coined to mean "the causal interactions between genes and their products which bring the phenotype into being" [1]. But it was not until the 1970s when the contemporary definition emerged as “a hereditable change in gene expression that occurred without a change in the DNA sequence” [2]. Broadly speaking, as it applies to modern cancer biology, epigenetics now refers to regulatory mechanisms of DNA transcription that affect gene expression of which DNA methylation is the most widely studied. The relative role of epigenetics in cancer has been attributed to the observation in 1983 by two laboratories that most cancer DNA has fewer methyl groups than non-cancer DNA [3-5]. In one of these studies, Feinberg and Vogelstein showed that DNA methylation was linked to tissue-specific gene silencing in cancer, by finding that a substantial proportion of CpG islands were methylated in normal tissues were unmethylated in cancer cells [3].

Citation analysis is a systematic approach for identifying scientific publications that have a high impact in the scientific or medical community measuring high-impact papers and how they have shaped scientific disciplines [6]. For this purpose, the Institute for Scientific Information collects citation counts for academic journals in the Science Citation Index. Although bibliometric analyses have been performed in the past on cancer and genomics, little is known about the most frequently cited articles specifically related to cancer epigenetics [6-10]. Therefore, the purpose of this study is to use citation count to identify those papers in the scientific literature that have made key contributions in the field of cancer epigenetics and identify key driving forces behind future investigations.

## Materials and methods

The Thomson Reuters Web of Science (WoS) database was used to query for citations of all articles relevant to cancer epigenetics. The basic search tool was selected, the keyword search for the topic to identify the articles of interest was specified as: “(epigenetic OR epigenomic OR methylation OR hypermethylation OR CpG island OR chromatic remodeling OR histone modification OR RNA interference OR gene silencing OR promoter regions OR chromatin assembly and disassembly OR liquid biopsy OR molecular OR biomolecular) AND (cancer OR neoplasm)”. The following search parameters were used: 1) articles published in the years 1980-2018 (since the word "epigenetics" was conceived in 1980); 2) all languages; 3) within the Science Citation Index Expanded. The results were carefully reviewed, and only those relevant to cancer epigenetics were selected. All review articles were excluded from the list. The top 100 articles by the number of citations that matched the search criteria were then further analyzed, and the title, first author, journal, and year of publication, number of citations, country, and the institution of origin were recorded. The articles retrieved were sorted in descending order in terms of times cited, and the number of citations per year was calculated.

## Results

**Figure 1:**
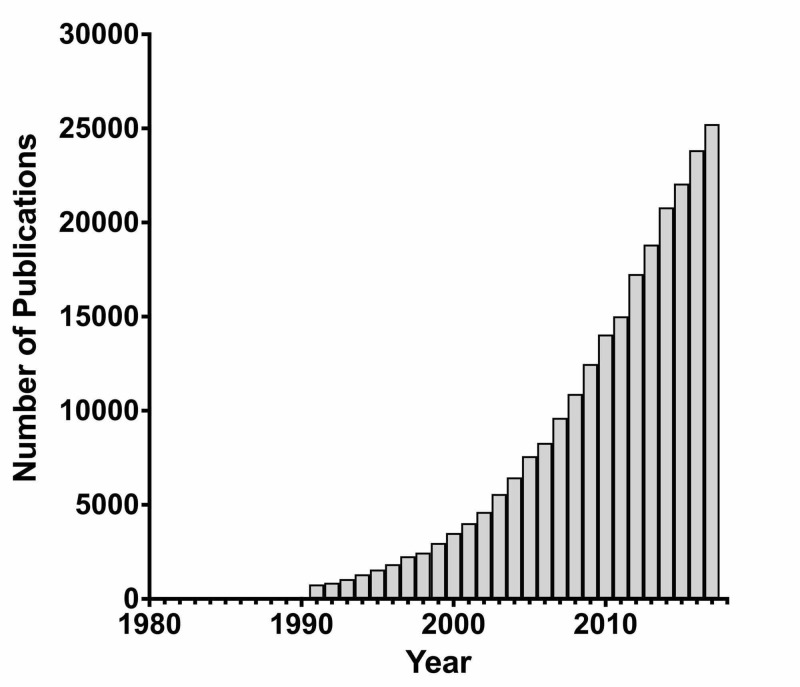
Number of publications per year retrieved from the Thomson Reuters Web of Science with the keyword search described in methods

Our query retrieved 234,679 papers (Figure 1).

The top 100 articles related to “cancer epigenetics” were identified by the number of times they were cited (Table 1).

**Table 1:** The top 100 most cited articles in cancer epigenetics ranked by number of times cited CY - number of citations per year; WoS - Web of Knowledge Citations corresponding to WoS rank are located in appendices.

WoS Rank	Authors	Year	Article title	Total number of citations	CY index	CY rank
1	Golub et al.	1999	Molecular classification of cancer: class discovery and class prediction by gene expression monitoring	7124	375	6
2	Alizadeh et al.	2000	Distinct types of diffuse large B-cell lymphoma identified by gene expression profiling	6045	336	13
3	Herman et al.	1996	Methylation-specific PCR: a novel PCR assay for methylation status of CpG islands	4600	209	26
4	Barski et al.	2007	High-resolution profiling of histone methylations in the human genome	3849	350	10
5	Hegi et al.	2005	MGMT gene silencing and benefit from temozolomide in glioblastoma	3394	261	21
6	Chin et al.	2008	Comprehensive genomic characterization defines human glioblastoma genes and core pathways	3359	336	12
7	Cerami et al.	2012	The cBio cancer genomics portal: an open platform for exploring multidimensional cancer genomics data	3354	559	2
8	Stupp et al.	2009	Effects of radiotherapy with concomitant and adjuvant temozolomide versus radiotherapy alone on survival in glioblastoma in a randomised phase III study: 5-year analysis of the EORTC-NCIC trial	3252	361	8
9	Muzny et al.	2012	Comprehensive molecular characterization of human colon and rectal cancer	3157	526	3
10	Verhaak et al.	2010	Integrated genomic analysis identifies clinically relevant subtypes of glioblastoma characterized by abnormalities in PDGFRA, IDH1, EGFR, and NF1	2891	361	7
11	Bell et al.	2011	Integrated genomic analyses of ovarian carcinoma	2725	389	5
12	Gupta et al.	2010	Long non-coding RNA HOTAIR reprograms chromatin state to promote cancer metastasis	2637	330	15
13	Forner et al.	2012	Hepatocellular carcinoma	2427	405	4
14	Travis et al.	2011	International association for the study of lung cancer/American Thoracic Society/European Respiratory Society International Multidisciplinary Classification of lung adenocarcinoma	2225	318	17
15	Yanaihara et al.	2006	Unique microRNA molecular profiles in lung cancer diagnosis and prognosis	2169	181	27
16	Curtis et al.	2012	The genomic and transcriptomic architecture of 2,000 breast tumours reveals novel subgroups	1911	319	16
17	Neve et al.	2006	A collection of breast cancer cell lines for the study of functionally distinct cancer subtypes	1828	152	30
18	Nielsen et al.	2004	Immunohistochemical and clinical characterization of the basal-like subtype of invasive breast carcinoma	1731	124	42
19	Hammerman et al.	2012	Comprehensive genomic characterization of squamous cell lung cancers	1731	289	19
20	Toyota et al.	1999	CpG island methylator phenotype in colorectal cancer	1701	90	58
21	Takamizawa et al.	2004	Reduced expression of the let-7 microRNAs in human lung cancers in association with shortened postoperative survival	1695	121	43
22	Merlo et al.	1995	5' CpG island methylation is associated with transcriptional silencing of the tumour suppressor p16/CDKN2/MTS1 in human cancers	1671	73	72
23	Ley et al.	2013	Genomic and epigenomic landscapes of adult de novo acute myeloid leukemia	1656	331	14
24	Varambally et al.	2002	The polycomb group protein EZH2 is involved in progression of prostate cancer	1625	102	50
25	Bhattacharjee et al.	2001	Classification of human lung carcinomas by mRNA expression profiling reveals distinct adenocarcinoma subclasses	1624	96	52
26	Esteller et al.	2001	A gene hypermethylation profile of human cancer	1605	94	55
27	Meissner et al.	2008	Genome-scale DNA methylation maps of pluripotent and differentiated cells	1538	154	29
28	Zhang et al.	2007	microRNAs as oncogenes and tumor suppressors	1515	138	36
29	Kandoth et al.	2013	Mutational landscape and significance across 12 major cancer types	1506	301	18
30	Cameron et al.	1999	Synergy of demethylation and histone deacetylase inhibition in the re-expression of genes silenced in cancer	1473	78	66
31	Clark et al.	1994	High sensitivity mapping of methylated cytosines.	1464	61	80
32	Herman et al.	1998	Incidence and functional consequences of hMLH1 promoter hypermethylation in colorectal carcinoma	1455	73	71
33	Bass et al.	2014	Comprehensive molecular characterization of gastric adenocarcinoma	1403	351	9
34	Collisson et al.	2014	Comprehensive molecular profiling of lung adenocarcinoma	1372	343	11
35	Brennan et al.	2013	The somatic genomic landscape of glioblastoma	1367	273	20
36	Esteller et al.	2000	Inactivation of the DNA-repair gene MGMT and the clinical response of gliomas to alkylating agents	1360	76	69
37	Weinstein et al.	2013	The cancer genome atlas pan-cancer analysis project	1293	259	22
38	Weber et al.	2007	Distribution, silencing potential and evolutionary impact of promoter DNA methylation in the human genome	1289	117	47
39	Figueroa et al.	2010	Leukemic IDH1 and IDH2 mutations result in a hypermethylation phenotype, disrupt TET2 function, and impair hematopoietic differentiation	1280	160	28
40	Getz et al.	2013	Integrated genomic characterization of endometrial carcinoma	1273	255	23
41	Irizarry et al.	2009	The human colon cancer methylome shows similar hypo- and hypermethylation at conserved tissue-specific CpG island shores	1267	141	35
42	Herman et al.	1995	Inactivation of the CDKN2/p16/MTS1 gene is frequently associated with aberrant DNA methylation in all common human cancers.	1241	54	88
43	Narita et al.	2003	Rb-mediated heterochromatin formation and silencing of E2F target genes during cellular senescence	1238	83	64
44	Herman et al.	1994	Silencing of the VHL tumor-suppressor gene by DNA methylation in renal carcinoma.	1226	51	91
45	Swerdlow et al.	2016	The 2016 revision of the World Health Organization classification of lymphoid neoplasms	1201	601	1
46	Noushmehr et al.	2010	Identification of a CpG Island methylator phenotype that defines a distinct subgroup of glioma	1170	146	32
47	Kane et al.	1997	Methylation of the hMLH1 promoter correlates with lack of expression of hMLH1 in sporadic colon tumors and mismatch repair-defective human tumor cell lines	1164	55	84
48	Weisenberger et al.	2006	CpG island methylator phenotype underlies sporadic microsatellite instability and is tightly associated with BRAF mutation in colorectal cancer	1162	97	51
49	Weber et al.	2005	Chromosome-wide and promoter-specific analyses identify sites of differential DNA methylation in normal and transformed human cells	1087	84	61
50	Fraga et al.	2005	Loss of acetylation at Lys16 and trimethylation at Lys20 of histone H4 is a common hallmark of human cancer	1060	82	65
51	Fabbri et al.	2007	MicroRNA-29 family reverts aberrant methylation in lung cancer by targeting DNA methyltransferases 3A and 3B	1041	95	54
52	Orom et al.	2010	Long noncoding RNAs with enhancer-like function in human cells	1041	130	39
53	Kleer et al.	2003	EZH2 is a marker of aggressive breast cancer and promotes neoplastic transformation of breast epithelial cells	1014	68	77
54	Weinstein et al.	2014	Comprehensive molecular characterization of urothelial bladder carcinoma	1014	254	24
55	Jahr et al.	2001	DNA fragments in the blood plasma of cancer patients: quantitations and evidence for their origin from apoptotic and necrotic cells	1005	59	82
56	Hudson et al.	2010	International network of cancer genome projects	1000	125	40
57	Costello et al.	2000	Aberrant CpG-island methylation has non-random and tumour-type-specific patterns	994	55	85
58	Gaudet et al.	2003	Induction of tumors in mice by genomic hypomethylation	993	66	78
59	Sharma et al.	2010	A Chromatin-mediated reversible drug-tolerant state in cancer cell subpopulations	968	121	44
60	Esteller et al.	1999	Inactivation of the DNA repair gene O-6-methylguanine-DNA methyltransferase by promoter hypermethylation is a common event in primary human neoplasia	956	50	92
61	Zuber et al.	2011	RNAi screen identifies Brd4 as a therapeutic target in acute myeloid leukaemia	941	134	37
62	Iorio et al.	2007	MicroRNA signatures in human ovarian cancer	937	85	60
63	Dweep et al.	2011	miRWalk - database: prediction of possible miRNA binding sites by "walking" the genes of three genomes	936	134	38
64	Comijn et al.	2001	The two-handed E box binding zinc finger protein SIP1 downregulates E-cadherin and induces invasion	933	55	86
65	Issa et al.	1994	Methylation of the oestrogen receptor CpG island links aging and neoplasia in human colon.	925	39	98
66	Kosaka et al.	2010	Secretory mechanisms and intercellular transfer of microRNAs in living cells	920	115	48
67	Saito et al.	2006	Specific activation of microRNA-127 with downregulation of the proto-oncogene BCL6 by chromatin-modifying drugs in human cancer cells	917	76	67
68	Carroll et al.	2006	Genome-wide analysis of estrogen receptor binding sites	911	76	68
69	Valk et al.	2004	Prognostically useful gene-expression profiles in acute myeloid leukemia	907	65	79
70	Weinstein et al.	1997	An information-intensive approach to the molecular pharmacology of cancer	906	43	95
71	Kantarjian et al.	2006	Decitabine improves patient outcomes in myelodysplastic syndromes - resuits of a phase III randomized study	899	75	70
72	Houseman et al.	2012	DNA methylation arrays as surrogate measures of cell mixture distribution	896	149	31
73	Patel et al.	2014	Single-cell RNA-seq highlights intratumoral heterogeneity in primary glioblastoma	896	224	25
74	West et al.	2001	Predicting the clinical status of human breast cancer by using gene expression profiles	891	52	90
75	Turchinovich et al.	2011	Characterization of extracellular circulating microRNA	874	125	41
76	McCabe et al.	2012	EZH2 inhibition as a therapeutic strategy for lymphoma with EZH2-activating mutations	861	144	33
77	Dammann et al.	2000	Epigenetic inactivation of a RAS association domain family protein from the lung tumour suppressor locus 3p21.3	855	48	94
78	Turcan et al.	2012	IDH1 mutation is sufficient to establish the glioma hypermethylator phenotype	854	142	34
79	Rhee et al.	2002	DNMT1 and DNMT3b cooperate to silence genes in human cancer cells	852	53	89
80	Lapointe et al.	2004	Gene expression profiling identifies clinically relevant subtypes of prostate cancer	849	61	81
81	Eckhardt et al.	2006	DNA methylation profiling of human chromosomes 6, 20 and 22	847	71	73
82	Bos et al.	2009	Genes that mediate breast cancer metastasis to the brain	847	94	56
83	Iliopoulos et al.	2009	An epigenetic switch involving NF-kappa B, lin28, let-7 microRNA, and IL6 links inflammation to cell transformation	845	94	57
84	Bracken et al.	2006	Genome-wide mapping of polycomb target genes unravels their roles in cell fate transitions	842	70	74
85	Campo et al.	2011	The 2008 WHO classification of lymphoid neoplasms and beyond: evolving concepts and practical applications	825	118	45
86	Chapman et al.	2011	Initial genome sequencing and analysis of multiple myeloma	824	118	46
87	Murakami et al.	2006	Comprehensive analysis of microRNA expression patterns in hepatocellular carcinoma and non-tumorous tissues	815	68	76
88	Gregoretti et al.	2004	Molecular evolution of the histone deacetylase family: Functional implications of phylogenetic analysis	806	58	83
89	Li et al.	2002	Causal relationship between the loss of RUNX3 expression and gastric cancer	805	50	93
90	Ng et al.	2009	Differential expression of microRNAs in plasma of patients with colorectal cancer: a potential marker for colorectal cancer screening	794	88	59
91	Bibikova et al.	2011	High density DNA methylation array with single CpG site resolution	762	109	49
92	Yap et al.	2010	Molecular interplay of the noncoding RNA ANRIL and methylated histone H3 lysine 27 by polycomb CBX7 in transcriptional silencing of INK4a	760	95	53
93	Suzuki et al.	2004	Epigenetic inactivation of SFRP genes allows constitutive WNT signaling in colorectal cancer	758	54	87
94	Esteller et al.	2000	Promoter hypermethylation and BRCA1 inactivation in sporadic breast and ovarian tumors	756	42	96
95	Schlesinger et al.	2007	Polycomb-mediated methylation on Lys27 of histone H3 pre-marks genes for de novo methylation in cancer	748	68	75
96	Shimono et al.	2009	Downregulation of miRNA-200c links breast cancer stem cells with normal stem cells	745	83	62
97	Doi et al.	2009	Differential methylation of tissue- and cancer-specific CpG island shores distinguishes human induced pluripotent stem cells, embryonic stem cells and fibroblasts	744	83	63
98	Esteller et al.	1999	Detection of aberrant promoter hypermethylation of tumor suppressor genes in serum DNA from non-small cell lung cancer patients	741	39	97
99	Belinsky et al.	1998	Aberrant methylation of p16[INK4a] is an early event in lung cancer and a potential biomarker for early diagnosis	723	36	99
100	Rainier et al.	1993	Relaxation of imprinted genes in human cancer.	720	29	100

The articles on this top 100 list were cited between 7,124 times (article rank 1) and 720 times (article rank 100). Collectively, the top 100 articles have been cited 147,083 times with a median of 1,050 for each paper, and an interquartile range of 871 - 1610. The oldest article on the top 100 list was from 1993, and the most recent from 2016. In the period 1980-2018, the two most prolific years were 2006 and 2010, with nine articles each among the top 100 most cited articles. In terms of the number of citations per year, the top article had been cited 375 times per year (CY rank number 6). Likewise, the bottom article has been cited 29 times per year (CY rank number 100). A graph of time vs. publication output (Figure 1) indicates that the field of cancer epigenetics has had publications in the range 1994-2014. The most productive decade was from 2000 to 2009, producing 49 papers in the Top 100 (Table 2).

**Table 2:** Decade of publication of top 100 in cancer epigenetics

Decade of publication	No. of articles (n=100)
1970-1979	0
1980-1989	0
1990-1999	13
2000-2009	49
2010-2019	27

The top 100 most cited articles were published in 28 different journals, with the journal Nature contributing the most studies with 22 articles (Table 3).

**Table 3:** Journals of origin

Rank	Journal	No. of articles (n=100)
1	Nature	22
2	Nature Genetics	15
3	Proceedings of the National Academy of Sciences of the United States of America	10
4	Cancer Research	8
4	Cell	8
5	Cancer Cell	6
6	New England Journal of Medicine	4
6	Science	4
7	Blood	2
7	Molecular Cell	2
7	Nucleic Acids Research	2
8	BMC Bioinformatics	1
8	Cancer	1
8	Cancer Discovery	1
8	Clinical Cancer Research	1
8	Developmental Biology	1
8	Genes Development	1
8	Genomics	1
8	Gut	1
8	Journal of the National Cancer Institute	1
8	Journal of Biological Chemistry	1
8	Journal of Biomedical Informatics	1
8	Journal of Molecular Biology	1
8	Journal of Thoracic Oncology	1
8	Lancet	1
8	Lancet Oncology	1
8	Nature Medicine	1
8	Oncogene	1

Seventy-eight percent of the top 100 most cited papers originated in the United States (n=78). The next five countries with the highest number of articles were Germany (n=4), Switzerland (n=4), Japan (n=3), Spain (n=2), and United Kingdom (n=2). Australia, Belgium, Denmark, Israel, Netherlands, China, and South Korea had one article, each among the top 100. Among the 100 most cited papers, there were a total of 77 unique first authors. Collectively, the two authors with the largest number of articles on the top 100 list were Baylin SB and Herman JG with 26 and 20 papers, respectively (Table 4). The next five authors that followed were Getz G, Laird PW, Meyerson M, Sander C, and Weisenberger DJ, each with 13, 12, 12, 12, and 12 articles, respectively.

**Table 4:** Top five authors appearing in top 100 list

Rank	Author	No. of articles (n=100)
1	Baylin SB	26
2	Herman JG	20
3	Getz G	13
4	Laird PW	12
4	Meyerson M	12
4	Sander C	12
4	Weisenberger DJ	12
5	Ding L	11
5	Hayes DN	11
5	Lander ES	11
5	Perou CM	11

Among the top 100 cited papers, there were three clinical trials, two guidelines or society-based recommendations, 18 cancer classifications, 11 articles related to research tools or methods, 55 articles related to epigenetic cancer mechanism, nine papers related to epigenetic cancer markers/screening/diagnosis and five papers related to epigenetics and cancer treatment (Table 5).

**Table 5:** The top 100 most cited articled in cancer epigenetics categorized by review, clinical trials, guidelines of society-based recommendations, classifications, research tools/methods, epigenetic mechanisms, epigenetic markers/screening/diagnosis, and epigenetic cancer treatment WoS - Web of Knowledge Citations corresponding to WoS rank are located in appendices.

WoS citation rank	Authors	Year	Article title	Total number of citations
CLINICAL TRIALS (n=3)				
5	Hegi et al.	2005	MGMT gene silencing and benefit from temozolomide in glioblastoma	3394
8	Stupp et al.	2009	Effects of radiotherapy with concomitant and adjuvant temozolomide versus radiotherapy alone on survival in glioblastoma in a randomised phase III study: 5-year analysis of the EORTC-NCIC trial	3252
71	Kantarjian et al.	2006	Decitabine improves patient outcomes in myelodysplastic syndromes - Resuits of a Phase III randomized study	899
GUIDELINES OR SOCIETY-BASED RECOMMENDATIONS (n=3)				
14	Travis et al.	2011	International association for the study of lung cancer/American Thoracic Society/European Respiratory Society International Multidisciplinary Classification of lung adenocarcinoma	2225
56	Hudson et al.	2010	International network of cancer genome projects	1000
CLASSIFICATIONS (n=18)				
1	Golub et al.	1999	Molecular classification of cancer: class discovery and class prediction by gene expression monitoring	7124
2	Alizadeh et al.	2000	Distinct types of diffuse large B-cell lymphoma identified by gene expression profiling	6045
10	Verhaak et al.	2010	Integrated genomic analysis identifies clinically relevant subtypes of glioblastoma characterized by abnormalities in PDGFRA, IDH1, EGFR, and NF1	2891
16	Curtis et al.	2012	The genomic and transcriptomic architecture of 2,000 breast tumours reveals novel subgroups	1911
17	Neve et al.	2006	A collection of breast cancer cell lines for the study of functionally distinct cancer subtypes	1828
18	Nielsen et al.	2004	Immunohistochemical and clinical characterization of the basal-like subtype of invasive breast carcinoma	1731
20	Toyota et al.	1999	CpG island methylator phenotype in colorectal cancer	1701
23	Ley et al.	2013	Genomic and epigenomic landscapes of adult de novo acute myeloid leukemia	1656
25	Bhattacharjee et al.	2001	Classification of human lung carcinomas by mRNA expression profiling reveals distinct adenocarcinoma subclasses	1624
29	Kandoth et al.	2013	Mutational landscape and significance across 12 major cancer types	1506
35	Brennan et al.	2013	The somatic genomic landscape of glioblastoma	1367
39	Figueroa et al.	2010	Leukemic IDH1 and IDH2 mutations result in a hypermethylation phenotype, disrupt TET2 function, and impair hematopoietic differentiation	1280
45	Swerdlow et al.	2016	The 2016 revision of the World Health Organization classification of lymphoid neoplasms	1201
46	Noushmehr et al.	2010	Identification of a CpG island methylator phenotype that defines a distinct subgroup of glioma	1170
48	Weisenberger et al.	2006	CpG island methylator phenotype underlies sporadic microsatellite instability and is tightly associated with BRAF mutation in colorectal cancer	1162
78	Turcan et al.	2012	IDH1 mutation is sufficient to establish the glioma hypermethylator phenotype	854
80	Lapointe et al.	2004	Gene expression profiling identifies clinically relevant subtypes of prostate cancer	849
85	Campo et al.	2011	The 2008 WHO classification of lymphoid neoplasms and beyond: evolving concepts and practical applications	825
RESEARCH TOOLS/METHODS (n=11)				
3	Herman et al.	1996	Methylation-specific PCR: a novel PCR assay for methylation status of CpG islands	4600
4	Barski et al.	2007	High-resolution profiling of histone methylations in the human genome	3849
7	Cerami et al.	2012	The cBio cancer genomics portal: an open platform for exploring multidimensional cancer genomics data	3354
31	Clark et al.	1994	High sensitivity mapping of methylated cytosines.	1464
37	Weinstein et al.	2013	The cancer genome Atlas Pan-Cancer analysis project	1293
49	Weber et al.	2005	Chromosome-wide and promoter-specific analyses identify sites of differential DNA methylation in normal and transformed human cells	1087
63	Dweep et al.	2011	miRWalk - database: prediction of possible miRNA binding sites by "walking" the genes of three genomes	936
70	Weinstein et al.	1997	An information-intensive approach to the molecular pharmacology of cancer	906
72	Houseman et al.	2012	DNA methylation arrays as surrogate measures of cell mixture distribution	896
75	Turchinovich et al.	2011	Characterization of extracellular circulating microRNA	874
91	Bibikova et al.	2011	High density DNA methylation array with single CpG site resolution	762
EPIGENETIC CANCER MARKERS/SCREENING/DIAGNOSIS (n=9)				
15	Yanaihara et al.	2006	Unique microRNA molecular profiles in lung cancer diagnosis and prognosis	2169
21	Takamizawa et al.	2004	Reduced expression of the let-7 microRNAs in human lung cancers in association with shortened postoperative survival	1695
53	Kleer et al.	2003	EZH2 is a marker of aggressive breast cancer and promotes neoplastic transformation of breast epithelial cells	1014
55	Jahr et al.	2001	DNA fragments in the blood plasma of cancer patients: quantitations and evidence for their origin from apoptotic and necrotic cells	1005
69	Valk et al.	2004	Prognostically useful gene-expression profiles in acute myeloid leukemia	907
74	West et al.	2001	Predicting the clinical status of human breast cancer by using gene expression profiles	891
90	Ng et al.	2009	Differential expression of microRNAs in plasma of patients with colorectal cancer: a potential marker for colorectal cancer screening	794
98	Esteller et al.	1999	Detection of aberrant promoter hypermethylation of tumor suppressor genes in serum DNA from non-small cell lung cancer patients	741
99	Belinsky et al.	1998	Aberrant methylation of p16(INK4a) is an early event in lung cancer and a potential biomarker for early diagnosis	723
EPIGENETIC CANCER TREATMENT (n=5)				
5	Hegi et al.	2005	MGMT gene silencing and benefit from temozolomide in glioblastoma	3394
8	Stupp et al.	2009	Effects of radiotherapy with concomitant and adjuvant temozolomide versus radiotherapy alone on survival in glioblastoma in a randomised phase III study: 5-year analysis of the EORTC-NCIC trial	3252
61	Zuber et al.	2011	RNAi screen identifies Brd4 as a therapeutic target in acute myeloid leukaemia	941
71	Kantarjian et al.	2006	Decitabine improves patient outcomes in myelodysplastic syndromes - resuits of a phase III randomized study	899
76	McCabe et al.	2012	EZH2 inhibition as a therapeutic strategy for lymphoma with EZH2-activating mutations	861

## Discussion

In this study, we sought to identify the most cited 100 articles regarding cancer epigenetics, to gain insight into the history and future directions of this rapidly growing scientific field.

The article that received the most citations on the top 100 list was “Molecular classification of cancer: class discovery and class prediction by gene expression monitoring” [11]. This paper was cited 7,340 times, with an average of 408 citations per year since publication. At the time, the paper was notable for developing the first generalized approach for identifying new cancer classes by applying gene expression profiling to distinguish between acute myeloid leukemia (AML) versus acute lymphoblastic leukemia (ALL). This study marked the beginning of gene expression-based cancer therapy. Currently, the European LeukemiaNet classification in AML uses cytogenetic and molecular data to identify the AML prognostic groups [12-14].

Since the first epigenetic abnormality was identified in cancer cells in 1983, multiple advances led to improved knowledge in epigenetics and cancer [3-5]. DNA methylation has been defined as an example of epigenetic dysregulation in cancer, with both hypomethylation and hyper-methylation having significant roles. DNA hypomethylation can lead to gene activation, and it is linked to chromosomal instability [15, 16]. DNA hypermethylation has been associated with gene silencing as a tumor-suppressor silencing cancer mechanism given that it has been found when genes are rarely mutated but that are frequently DNA hypermethylated and silenced in cancer [17-20]. Histone modification is another epigenetic cancer-linked mechanism that controls chromatin structure [21, 22]. As a result, the detection of epigenetic changes, such as abnormal promoter CpG island DNA hypermethylation, has been studied as a potential biomarker strategy for assessing cancer risk, early detection, prognosis and predicting therapeutic responses [23, 24]. The list of potential marker genes, knowledge of their position in cancer progression, and the development of ever more sensitive epigenetic detection strategies, including nanotechnology approaches, are all expanding [25, 26]. All these landmark discoveries led to the elucidation of novel cancer biomolecular mechanisms, new scientific research tools, and the development of new epigenetic-based targeted therapeutic avenues. As a result of that, “The National Institutes of Health (NIH) Roadmap Epigenomics Mapping Consortium” is accelerating the understanding of epigenomics in human health and disease together with the ENCODE Project (ENCyclopedia Of DNA Elements) [27, 28]. The most immediate future of this new exciting scientific field includes the development of liquid biopsies, personalized medicine, and targeted therapies.

Although citation analysis is a useful tool with the potential benefit of insight into literature trends, it is not without limitations. Over half a century has passed since the Science Citation Index (SCI) was launched as the first systematic effort to track citations in the scientific literature [29]. We recognize that citation counts have inherent biases and that they are not purely quantifiable systems to rank papers by their impact in the scientific literature. In an attempt to control for some of these inherent and potential biases, we utilized the citations per year index in addition to the total number of citations per paper. Despite that, older publications have had a longer timespan to accumulate citations giving them a distinct advantage over newer and potentially more relevant studies. Lastly, one hundred is an arbitrary number since the landmark articles in epigenetic research did not accumulate enough citations such as the paper by Gama-Sosa, Slagel, Trewyn, et al. "The 5-methylcytosine content of DNA from human tumors" that only had 574 citations [30]. Although metrics such as citation counts do have flaws, in the current era, they also serve as one way to measure objectively impact of an article in the scientific community.

## Conclusions

In this study, the 100 most cited articles in cancer epigenetics were examined, and the contributions from various authors, specialties, and countries were identified. Cancer epigenetics is a rapidly growing scientific field impacting translational research in cancer screening, diagnosis, classification, prognosis, and targeted treatments. Recognition of important historical contributions to this field may guide future investigations.

## Appendices

References from Table 1 and Table 5

1. Golub TR, Slonim DK, Tamayo P, et al.: Molecular classification of cancer: class discovery and class prediction by gene expression monitoring. Science. 1999, 286:531-537. 10.1126/science.286.5439.531

2. Alizadeh AA, Eisen MB, Davis RE, et al.: Distinct types of diffuse large B-cell lymphoma identified by gene expression profiling. Nature. 2000, 403:503-511. 10.1038/35000501

3. Herman JG, Graff JR, Myohanen S, Nelkin BD, Baylin SB: Methylation-specific PCR: a novel PCR assay for methylation status of CpG islands. P Natl Acad Scad USA. 1996, 93:9821-9826. 10.1073/pnas.93.18.9821

4. Barski A, Cuddapah S, Cui K, et al.: High-resolution profiling of histone methylations in the human genome. Cell. 2007, 129:823-837. 10.1016/j.cell.2007.05.009

5. Hegi ME, Diserens AC, Gorlia T, et al.: MGMT gene silencing and benefit from temozolomide in glioblastoma. N Engl J Med. 2005, 352:997-1003. 10.1056/NEJMoa043331

6. Cancer Genome Atlas Research Network: Comprehensive genomic characterization defines human glioblastoma genes and core pathways. Nature. 2008, 455:1061-1068. 10.1038/nature07385

7. Cerami E, Gao J, Dogrusoz U, et al.: The cBio cancer genomics portal: an open platform for exploring multidimensional cancer genomics data. Cancer Discov. 2012, 2:401-404. 10.1158/2159-8290.CD-12-0095

8. Stupp R, Hegi ME, Mason WP, etal.: Effects of radiotherapy with concomitant and adjuvant temozolomide versus radiotherapy alone on survival in glioblastoma in a randomised phase III study: 5-year analysis of the EORTC-NCIC trial. Lancet Oncol. 2009, 10:459-466. 10.1016/S1470-2045(09)70025-7

9. Cancer Genome Atlas Network: Comprehensive molecular characterization of human colon and rectal cancer. Nature. 2012, 487:330-337. 10.1038/nature11252

10. Verhaak RG, Hoadley KA, Purdom E, et al.: Integrated genomic analysis identifies clinically relevant subtypes of glioblastoma characterized by abnormalities in PDGFRA, IDH1, EGFR, and NF1. Cancer Cell. 2010, 17:98-110. 10.1016/j.ccr.2009.12.020

11. Cancer Genome Atlas Research Network: Integrated genomic analyses of ovarian carcinoma. Nature. 2011, 474:609-615. 10.1038/nature10166

12. Gupta RA, Shah N, Wang KC, et al.: Long non-coding RNA HOTAIR reprograms chromatin state to promote cancer metastasis. Nature. 2010, 464:1071-1076. 10.1038/nature08975

13. Forner A, Llovet JM, Bruix J: Hepatocellular carcinoma. The Lancet. 2012, 379:1245-1255. 10.1016/s0140-6736(11)61347-0

14. Travis WD, Brambilla E, Noguchi M, et al.: International association for the study of lung cancer/american thoracic society/european respiratory society international multidisciplinary classification of lung adenocarcinoma. J Thorac Oncol. 2011, 6:244-285. 10.1097/JTO.0b013e318206a221

15. Yanaihara N, Caplen N, Bowman E, et al.: Unique microRNA molecular profiles in lung cancer diagnosis and prognosis. Cancer Cell. 2006, 9:189-198. 10.1016/j.ccr.2006.01.025

16. Curtis C, Shah SP, Chin SF, et al.: The genomic and transcriptomic architecture of 2,000 breast tumours reveals novel subgroups. Nature. 2012, 486:346-352. 10.1038/nature10983

17. Neve RM, Chin K, Fridlyand J, et al.: A collection of breast cancer cell lines for the study of functionally distinct cancer subtypes. Cancer Cell. 2006, 10:515-527. 10.1016/j.ccr.2006.10.008

18. Nielsen TO, Hsu FD, Jensen K, et al.: Immunohistochemical and clinical characterization of the basal-like subtype of invasive breast carcinoma. Clin Cancer Res. 2004, 10:5367-5374. 10.1158/1078-0432.CCR-04-0220

19. Cancer Genome Atlas Research Network: Comprehensive genomic characterization of squamous cell lung cancers. Nature. 2012, 489:519-525. 10.1038/nature11404

20. Toyota M, Ahuja N, Ohe-Toyota M, Herman JG, Baylin SB, Issa JP: CpG island methylator phenotype in colorectal cancer. P Natl Acad Scad USA. 1999, 96:8681-8686. 10.1073/pnas.96.15.8681

21. Takamizawa J, Konishi H, Yanagisawa K, et al.: Reduced expression of the let-7 microRNAs in human lung cancers in association with shortened postoperative survival. Cancer Res. 2004, 64:3753-3756. 10.1158/0008-5472.CAN-04-0637

22. Merlo A, Herman JG, Mao L, et al.: 5' CpG island methylation is associated with transcriptional silencing of the tumour suppressor p16/CDKN2/MTS1 in human cancers. Nat Med. 1995, 1:686-692. 10.1038/nm0795-686

23. Cancer Genome Atlas Research Network, Ley TJ, Miller C, et al.: Genomic and epigenomic landscapes of adult de novo acute myeloid leukemia. N Engl J Med. 2013, 368:2059-2074. 10.1056/NEJMoa1301689

24. Varambally S, Dhanasekaran SM, Zhou M, et al.: The polycomb group protein EZH2 is involved in progression of prostate cancer. Nature. 2002, 419:624-629. 10.1038/nature01075

25. Bhattacharjee A, Richards WG, Staunton J, et al.: Classification of human lung carcinomas by mRNA expression profiling reveals distinct adenocarcinoma subclasses. P Natl Acad Scad USA. 2001, 98:13790-13795. 10.1073/pnas.191502998

26. Esteller M, Corn PG, Baylin SB, Herman JG: A gene hypermethylation profile of human cancer. Cancer Res. 2001, 61:3225-3229.

27. Meissner A, Mikkelsen TS, Gu H, Wet al.: Genome-scale DNA methylation maps of pluripotent and differentiated cells. Nature. 2008, 454:766-770. 10.1038/nature07107

28. Zhang B, Pan X, Cobb GP, Anderson TA: microRNAs as oncogenes and tumor suppressors. Dev Biol. 2007, 302:1-12. 10.1016/j.ydbio.2006.08.028

29. Kandoth C, McLellan MD, Vandin F, et al.: Mutational landscape and significance across 12 major cancer types. Nature. 2013, 502:333-339. 10.1038/nature12634

30. Cameron EE, Bachman KE, Myohanen S, Herman JG, Baylin SB: Synergy of demethylation and histone deacetylase inhibition in the re-expression of genes silenced in cancer. Nat Genet. 1999, 21:103-107. 10.1038/5047

31. Clark SJ, Harrison J, Paul CL, Frommer M: High sensitivity mapping of methylated cytosines. Nucleic Acids Res. 1994, 22:2990-2997. 10.1093/nar/22.15.2990

32. Herman JG, Umar A, Polyak K, et al.: Incidence and functional consequences of hMLH1 promoter hypermethylation in colorectal carcinoma. P Natl Acad Scad USA. 1998, 95:6870-6875. 10.1073/pnas.95.12.6870

33. Cancer Genome Atlas Research Network: Comprehensive molecular characterization of gastric adenocarcinoma. Nature. 2014, 513:202-209. 10.1038/nature13480

34. Cancer Genome Atlas Research Network: Comprehensive molecular profiling of lung adenocarcinoma. Nature. 2014, 511:543-550. 10.1038/nature13385

35. Brennan CW, Verhaak RG, McKenna A, et al.: The somatic genomic landscape of glioblastoma. Cell. 2013, 155:462-477. 10.1016/j.cell.2013.09.034

36. Esteller M, Garcia-Foncillas J, Andion E, et al.: Inactivation of the DNA-repair gene MGMT and the clinical response of gliomas to alkylating agents. N Engl J Med. 2000, 343:1350-1354. 10.1056/NEJM200011093431901

37. Cancer Genome Atlas Research Network, Weinstein JN, Collisson EA, et al.: The Cancer Genome Atlas Pan-Cancer analysis project. Nat Genet. 2013, 45:1113-1120. 10.1038/ng.2764

38. Weber M, Hellmann I, Stadler MB, Ramos L, Paabo S, Rebhan M, Schubeler D: Distribution, silencing potential and evolutionary impact of promoter DNA methylation in the human genome. Nat Genet. 2007, 39:457-466. 10.1038/ng1990

39. Figueroa ME, Abdel-Wahab O, Lu C, et al.: Leukemic IDH1 and IDH2 mutations result in a hypermethylation phenotype, disrupt TET2 function, and impair hematopoietic differentiation. Cancer Cell. 2010, 18:553-567. 10.1016/j.ccr.2010.11.015

40. Cancer Genome Atlas Research Network, Kandoth C, Schultz N, et al.: Integrated genomic characterization of endometrial carcinoma. Nature. 2013, 497:67-73. 10.1038/nature12113

41. Irizarry RA, Ladd-Acosta C, Wen B, et al.: The human colon cancer methylome shows similar hypo- and hypermethylation at conserved tissue-specific CpG island shores. Nat Genet. 2009, 41:178-186. 10.1038/ng.298

42. Herman JG, Merlo A, Mao L, et al.: Inactivation of the Cdkn2/P16/Mts1 gene is frequently associated with aberrant DNA methylation in all common human cancers. Cancer Res. 1995, 55:4525-4530.

43. Narita M, Nunez S, Heard E, et al.: Rb-mediated heterochromatin formation and silencing of E2F target genes during cellular senescence. Cell. 2003, 113:703-716. 10.1016/s0092-8674(03)00401-x

44. Herman JG, Latif F, Weng Y, et al.: Silencing of the VHL tumor-suppressor gene by DNA methylation in renal carcinoma. P Natl Acad Scad USA. 1994, 91:9700-9704. 10.1073/pnas.91.21.9700

45. Swerdlow SH, Campo E, Pileri SA, et al.: The 2016 revision of the World Health Organization classification of lymphoid neoplasms. Blood. 2016, 127:2375-2390. 10.1182/blood-2016-01-643569

46. Noushmehr H, Weisenberger DJ, Diefes K, et al.: Identification of a CpG island methylator phenotype that defines a distinct subgroup of glioma. Cancer Cell. 2010, 17:510-522. 10.1016/j.ccr.2010.03.017

47. Kane MF, Loda M, Gaida GM, et al.: Methylation of the hMLH1 promoter correlates with lack of expression of hMLH1 in sporadic colon tumors and mismatch repair-defective human tumor cell lines. Cancer Res. 1997, 57:808-811.

48. Weisenberger DJ, Siegmund KD, Campan M, et al.: CpG island methylator phenotype underlies sporadic microsatellite instability and is tightly associated with BRAF mutation in colorectal cancer. Nat Genet. 2006, 38:787-793. 10.1038/ng1834

49. Weber M, Davies JJ, Wittig D, Oakeley EJ, Haase M, Lam WL, Schubeler D: Chromosome-wide and promoter-specific analyses identify sites of differential DNA methylation in normal and transformed human cells. Nat Genet. 2005, 37:853-862. 10.1038/ng1598

50. Fraga MF, Ballestar E, Villar-Garea A, et al.: Loss of acetylation at Lys16 and trimethylation at Lys20 of histone H4 is a common hallmark of human cancer. Nat Genet. 2005, 37:391-400. 10.1038/ng1531

51. Fabbri M, Garzon R, Cimmino A, et al.: MicroRNA-29 family reverts aberrant methylation in lung cancer by targeting DNA methyltransferases 3A and 3B. P Natl Acad Scad USA. 2007, 104:15805-15810. 10.1073/pnas.0707628104

52. Orom UA, Derrien T, Beringer M, et al.: Long noncoding RNAs with enhancer-like function in human cells. Cell. 2010, 143:46-58. 10.1016/j.cell.2010.09.001

53. Kleer CG, Cao Q, Varambally S, et al.: EZH2 is a marker of aggressive breast cancer and promotes neoplastic transformation of breast epithelial cells. P Natl Acad Scad USA. 2003, 100:11606-11611. 10.1073/pnas.1933744100

54. Cancer Genome Atlas Research Network: Comprehensive molecular characterization of urothelial bladder carcinoma. Nature. 2014, 507:315-322. 10.1038/nature12965

55. Jahr S, Hentze H, Englisch S, Hardt D, Fackelmayer FO, Hesch RD, Knippers R: DNA fragments in the blood plasma of cancer patients: Quantitations and evidence for their origin from apoptotic and necrotic cells. Cancer Res. 2001, 61:1659-1665.

56. International Cancer Genome Consortium, Hudson TJ, Anderson W, et al.: International network of cancer genome projects. Nature. 2010, 464:993-998. 10.1038/nature08987

57. Costello JF, Fruhwald MC, Smiraglia DJ, et al.: Aberrant CpG-island methylation has non-random and tumour-type-specific patterns. Nat Genet. 2000, 24:132-138. 10.1038/72785

58. Gaudet F, Hodgson JG, Eden A, et al.: Induction of tumors in mice by genomic hypomethylation. Science. 2003, 300:489-492. 10.1126/science.1083558

59. Sharma SV, Lee DY, Li B, et al.: A chromatin-mediated reversible drug-tolerant state in cancer cell subpopulations. Cell. 2010, 141:69-80. 10.1016/j.cell.2010.02.027

60. Esteller M, Hamilton SR, Burger PC, Baylin SB, Herman JG: Inactivation of the DNA repair gene O-6-methylguanine-DNA methyltransferase by promoter hypermethylation is a common event in primary human neoplasia. Cancer Res. 1999, 59:793-797.

61. Zuber J, Shi J, Wang E, et al.: RNAi screen identifies Brd4 as a therapeutic target in acute myeloid leukaemia. Nature. 2011, 478:524-528. 10.1038/nature10334

62. Iorio MV, Visone R, Di Leva G, et al.: MicroRNA signatures in human ovarian cancer. Cancer Res. 2007, 67:8699-8707. 10.1158/0008-5472.CAN-07-1936

63. Dweep H, Sticht C, Pandey P, Gretz N: miRWalk--database: prediction of possible miRNA binding sites by "walking" the genes of three genomes. J Biomed Inform. 2011, 44:839-847. 10.1016/j.jbi.2011.05.002

64. Comijn J, Berx G, Vermassen P, et al.: The two-handed E box binding zinc finger protein SIP1 downregulates E-cadherin and induces invasion. Mol Cell. 2001, 7:1267-1278. 10.1016/s1097-2765(01)00260-x

65. Issa JP, Ottaviano YL, Celano P, Hamilton SR, Davidson NE, Baylin SB: Methylation of the oestrogen receptor CpG island links ageing and neoplasia in human colon. Nat Genet. 1994, 7:536-540. 10.1038/ng0894-536

66. Kosaka N, Iguchi H, Yoshioka Y, Takeshita F, Matsuki Y, Ochiya T: Secretory mechanisms and intercellular transfer of microRNAs in living cells. J Biol Chem. 2010, 285:17442-17452. 10.1074/jbc.M110.107821

67. Saito Y, Liang G, Egger G, Friedman JM, Chuang JC, Coetzee GA, Jones PA: Specific activation of microRNA-127 with downregulation of the proto-oncogene BCL6 by chromatin-modifying drugs in human cancer cells. Cancer Cell. 2006, 9:435-443. 10.1016/j.ccr.2006.04.020

68. Carroll JS, Meyer CA, Song J, et al.: Genome-wide analysis of estrogen receptor binding sites. Nat Genet. 2006, 38:1289-1297. 10.1038/ng1901

69. Valk PJ, Verhaak RG, Beijen MA, et al.: Prognostically useful gene-expression profiles in acute myeloid leukemia. N Engl J Med. 2004, 350:1617-1628. 10.1056/NEJMoa040465

70. Weinstein JN, Myers TG, O'Connor PM, et al.: An information-intensive approach to the molecular pharmacology of cancer. Science. 1997, 275:343-349. 10.1126/science.275.5298.343

71. Kantarjian H, Issa JP, Rosenfeld CS, et al.: Decitabine improves patient outcomes in myelodysplastic syndromes: results of a phase III randomized study. Cancer. 2006, 106:1794-1803. 10.1002/cncr.21792

72. Houseman EA, Accomando WP, Koestler DC, et al.: DNA methylation arrays as surrogate measures of cell mixture distribution. BMC Bioinform. 2012, 13:86. 10.1186/1471-2105-13-86

73. Patel AP, Tirosh I, Trombetta JJ, et al.: Single-cell RNA-seq highlights intratumoral heterogeneity in primary glioblastoma. Science. 2014, 344:1396-1401. 10.1126/science.1254257

74. West M, Blanchette C, Dressman H, et al.: Predicting the clinical status of human breast cancer by using gene expression profiles. P Natl Acad Scad USA. 2001, 98:11462-11467. 10.1073/pnas.201162998

75. Turchinovich A, Weiz L, Langheinz A, Burwinkel B: Characterization of extracellular circulating microRNA. Nucleic Acids Res. 2011, 39:7223-7233. 10.1093/nar/gkr254

76. McCabe MT, Ott HM, Ganji G, et al.: EZH2 inhibition as a therapeutic strategy for lymphoma with EZH2-activating mutations. Nature. 2012, 492:108-112. 10.1038/nature11606

77. Dammann R, Li C, Yoon JH, Chin PL, Bates S, Pfeifer GP: Epigenetic inactivation of a RAS association domain family protein from the lung tumour suppressor locus 3p21.3. Nat Genet. 2000, 25:315-319. 10.1038/77083

78. Turcan S, Rohle D, Goenka A, et al.: IDH1 mutation is sufficient to establish the glioma hypermethylator phenotype. Nature. 2012, 483:479-483. 10.1038/nature10866

79. Rhee I, Bachman KE, Park BH, et al.: DNMT1 and DNMT3b cooperate to silence genes in human cancer cells. Nature. 2002, 416:552-556. 10.1038/416552a

80. Lapointe J, Li C, Higgins JP, et al.: Gene expression profiling identifies clinically relevant subtypes of prostate cancer. P Natl Acad Scad USA. 2004, 101:811-816. 10.1073/pnas.0304146101

81. Eckhardt F, Lewin J, Cortese R, et al.: DNA methylation profiling of human chromosomes 6, 20 and 22. Nat Genet. 2006, 38:1378-1385. 10.1038/ng1909

82. Bos PD, Zhang XH, Nadal C, et al.: Genes that mediate breast cancer metastasis to the brain. Nature. 2009, 459:1005-1009. 10.1038/nature08021

83. Iliopoulos D, Hirsch HA, Struhl K: An epigenetic switch involving NF-kappaB, Lin28, Let-7 MicroRNA, and IL6 links inflammation to cell transformation. Cell. 2009, 139:693-706. 10.1016/j.cell.2009.10.014

84. Bracken AP, Dietrich N, Pasini D, Hansen KH, Helin K: Genome-wide mapping of Polycomb target genes unravels their roles in cell fate transitions. Genes Dev. 2006, 20:1123-1136. 10.1101/gad.381706

85. Campo E, Swerdlow SH, Harris NL, Pileri S, Stein H, Jaffe ES: The 2008 WHO classification of lymphoid neoplasms and beyond: evolving concepts and practical applications. Blood. 2011, 117:5019-5032. 10.1182/blood-2011-01-293050

86. Chapman MA, Lawrence MS, Keats JJ, et al.: Initial genome sequencing and analysis of multiple myeloma. Nature. 2011, 471:467-472. 10.1038/nature09837

87. Murakami Y, Yasuda T, Saigo K, Urashima T, Toyoda H, Okanoue T, Shimotohno K: Comprehensive analysis of microRNA expression patterns in hepatocellular carcinoma and non-tumorous tissues. Oncogene. 2006, 25:2537-2545. 10.1038/sj.onc.1209283

88. Gregoretti IV, Lee YM, Goodson HV: Molecular evolution of the histone deacetylase family: functional implications of phylogenetic analysis. J Mol Biol. 2004, 338:17-31. 10.1016/j.jmb.2004.02.006

89. Li QL, Ito K, Sakakura C, et al.: Causal relationship between the loss of RUNX3 expression and gastric cancer. Cell. 2002, 109:113-124. 10.1016/S0092-8674(02)00690-6

90. Ng EK, Chong WW, Jin H, et al.: Differential expression of microRNAs in plasma of patients with colorectal cancer: a potential marker for colorectal cancer screening. Gut. 2009, 58:1375-1381. 10.1136/gut.2008.167817

91. Bibikova M, Barnes B, Tsan C, et al.: High density DNA methylation array with single CpG site resolution. Genomics. 2011, 98:288-295. 10.1016/j.ygeno.2011.07.007

92. Yap KL, Li S, Munoz-Cabello AM, Raguz S, Zeng L, Mujtaba S, Gil J, Walsh MJ, Zhou MM: Molecular interplay of the noncoding RNA ANRIL and methylated histone H3 lysine 27 by polycomb CBX7 in transcriptional silencing of INK4a. Mol Cell. 2010, 38:662-674. 10.1016/j.molcel.2010.03.021

93. Suzuki H, Watkins DN, Jair KW, et al.: Epigenetic inactivation of SFRP genes allows constitutive WNT signaling in colorectal cancer. Nat Genet. 2004, 36:417-422. 10.1038/ng1330

94. Esteller M, Silva JM, Dominguez G, et al.: Promoter hypermethylation and BRCA1 inactivation in sporadic breast and ovarian tumors. J Natl Cancer Inst. 2000, 92:564-569. 10.1093/jnci/92.7.564

95. Schlesinger Y, Straussman R, Keshet I, et al.: Polycomb-mediated methylation on Lys27 of histone H3 pre-marks genes for de novo methylation in cancer. Nat Genet. 2007, 39:232-236. 10.1038/ng1950

96. Shimono Y, Zabala M, Cho RW, et al.: Downregulation of miRNA-200c links breast cancer stem cells with normal stem cells. Cell. 2009, 138:592-603. 10.1016/j.cell.2009.07.011

97. Doi A, Park IH, Wen B, et al.: Differential methylation of tissue- and cancer-specific CpG island shores distinguishes human induced pluripotent stem cells, embryonic stem cells and fibroblasts. Nat Genet. 2009, 41:1350-1353. 10.1038/ng.471

98. Esteller M, Sanchez-Cespedes M, Rosell R, Sidransky D, Baylin SB, Herman JG: Detection of aberrant promoter hypermethylation of tumor suppressor genes in serum DNA from non-small cell lung cancer patients. Cancer Res. 1999, 59:67-70.

99. Belinsky SA, Nikula KJ, Palmisano WA, et al.: Aberrant methylation of p16(INK4a) is an early event in lung cancer and a potential biomarker for early diagnosis. P Natl Acad Scad USA. 1998, 95:11891-11896. 10.1073/pnas.95.20.11891

100. Rainier S, Johnson LA, Dobry CJ, Ping AJ, Grundy PE, Feinberg AP: Relaxation of imprinted genes in human cancer. Nature. 1993, 362:747-749. 10.1038/362747a0
